# Effects of Acupuncture Treatment Alone and in Combination With Japanese Kampo Medicines on Reduced Dietary Intake During Hospitalization: A Single-Center Case Series

**DOI:** 10.7759/cureus.85996

**Published:** 2025-06-14

**Authors:** Naoya Mitani, Masayuki Kashima, Satoshi Hayano, Kenichiro Tokunaga, Yuki Toyama

**Affiliations:** 1 Department of Internal Medicine, Japanese Red Cross Kumamoto Hospital, Kumamoto, JPN; 2 Department of General Medicine, Japanese Red Cross Kumamoto Hospital, Kumamoto, JPN

**Keywords:** acupuncture, enteral nutrition, hospitalization, japanese kampo medicine, malnutrition

## Abstract

Background: Malnutrition leads to various consequences, including prolonged hospital stays and an increased risk of readmission. Insufficient dietary intake often necessitates interventions such as parenteral or enteral nutrition, further extending the duration of hospitalization. Studies suggest that acupuncture may enhance appetite; however, its direct impact on dietary intake remains unclear. In this study, we aimed to evaluate the effects of acupuncture alone and in combination with Japanese Kampo medicines on reduced dietary intake and related blood parameters to clarify this relationship.

Methods: This single-center, retrospective observational study was conducted at the Japanese Red Cross Kumamoto Hospital, an acute care facility in Japan, and included all hospitalized patients who received acupuncture treatment for reduced dietary intake from February 1, 2020, to January 31, 2023. Reduced dietary intake was defined as an average intake of less than 50% during the seven days prior to the initiation of acupuncture treatment. The treatment sessions lasted 20-30 minutes, with interventions performed once daily, five times per week (Monday to Friday, excluding Japanese holidays). Japanese Kampo medicines were prescribed by attending physicians in the course of routine medical care. The rate of daily change in dietary intake percentage from seven days before to seven days after acupuncture treatment, changes in blood parameters (total lymphocyte count, hemoglobin, albumin, and prealbumin levels), and treatment safety were evaluated. The change in dietary intake percentage before and after acupuncture treatment was analyzed using a paired t-test.

Results: Of 80 patients who received acupuncture during the study period, 64 were ultimately included in the analysis. Of these, 21 (33%) were male patients and 43 (67%) were female patients, with a mean age of 75.0 ± 12.7 years. The daily dietary intake rate increased from -1.08% before acupuncture to 1.93% after treatment. Similar trends were observed for individual meals, with breakfast intake rising from -1.46% to 2.09%, lunch from -1.67% to 1.53%, and dinner from -0.70% to 2.22%. The cause of reduced dietary intake was not clearly identified. Rikkunshito (TJ-43) was the most commonly co-administered Japanese Kampo medicine (31.7%). The most frequently used acupoints included ST36 (54 patients), SP6 (35 patients), CV12 (33 patients), SP9 (29 patients), SP3 (26 patients), and KI3 (20 patients). No significant adverse events were observed.

Conclusions: Dietary intake significantly improved following acupuncture treatment alone and in combination with Japanese Kampo medicines. These findings suggest that the observed improvements in dietary intake may be attributable to these interventions rather than to natural recovery. There were limitations to the study methods, and future studies with prospective, high-quality research designs will be necessary.

## Introduction

Hospital malnutrition - defined as undernutrition that develops during hospitalization - is significantly associated with adverse clinical outcomes, including prolonged length of stay (LOS), increased morbidity, and elevated in-hospital mortality. It also contributes to rising healthcare costs. Reported prevalence rates vary widely, ranging from 11% to 69%, depending on geographic region and medical specialty [1,2].

A primary cause of hospital malnutrition is inadequate dietary intake, which often necessitates interventions such as central parenteral nutrition or nasogastric tube feeding. These measures may further extend hospitalization and increase the risk of readmission [3]. For example, a study conducted in a high-volume Internal Medicine and Gastroenterology Department in Italy reported that 52.3% of hospitalized patients were at nutritional risk at admission, based on the Nutritional Risk Score-2002. Additionally, 38.7% met the diagnostic criteria for malnutrition established by the European Society for Clinical Nutrition and Metabolism (ESPEN). Malnourished patients in this study experienced significantly longer hospital stays compared to their well-nourished counterparts [4].

Despite evidence from high-quality randomized controlled trials indicating that nutritional therapy can reduce morbidity and other complications related to malnutrition in certain patients, enhancing dietary intake in the hospital setting remains a persistent challenge. Although hospital malnutrition is reported to affect 20-50% of patients in acute care, only a minority receive nutritional interventions. Referral rates to registered dietitians remain low, ranging from 15% to 36% [5]. These findings suggest that nutritional management is often under-recognized and inadequately prioritized in clinical practice.

One reason for this ongoing challenge is that nutritional interventions, although clinically effective under controlled conditions, may not fully address the multifactorial barriers to adequate intake in real-world hospital environments. These barriers include disease severity, inflammation, gastrointestinal symptoms, psychological distress, lack of appetite, mealtime interruptions, and a lack of individualized care. As Schuetz et al. [6] highlighted in their review, effective nutritional management requires not only the prescription of nutrition support but also consistent implementation, patient engagement, and interdisciplinary collaboration. In many hospital settings, particularly in acute care, these components may be insufficiently integrated, limiting the impact of nutritional therapy on actual dietary intake.

Despite its clinical significance, the optimal use of individualized nutritional therapy for the prevention and treatment of hospital malnutrition remains unclear, including key factors such as the timing and duration of intervention [6]. Therefore, there is an urgent need to explore and implement novel strategies to enhance dietary intake and mitigate the burden of malnutrition among hospitalized patients.

Acupuncture has been reported to enhance appetite in patients with advanced cancer [7] and improve delayed gastric emptying in critically ill patients undergoing mechanical ventilation [8]. Studies suggest that acupuncture may modulate gastrointestinal motility by influencing the autonomic nervous system. For example, electroacupuncture at PC6 has been shown to reduce transient lower esophageal sphincter relaxations and improve gastric myoelectrical activity. Clinical trials in intensive care settings have also reported that acupuncture at specific points (e.g., PC6, ST36) may reduce gastric residual volumes, enhance nutrient delivery, and decrease reliance on prokinetic drugs [9]. While these findings suggest the potential role of acupuncture in managing gastrointestinal symptoms and improving nutrient absorption, previous research has largely focused on surrogate outcomes such as gastric emptying, nausea, or caloric targets. To date, no studies have evaluated the direct impact of acupuncture on actual dietary intake percentages or its relationship with objective nutritional biomarkers in general hospitalized patients.

In parallel, certain Japanese Kampo medicines have traditionally been used to enhance gastrointestinal function and appetite; however, their combined effects with acupuncture on nutritional status during hospitalization have not been sufficiently explored.

The aim of this study was to examine the changes in daily dietary intake rate before and after interventions with acupuncture alone and in combination with Japanese Kampo medicines in order to clarify their potential role in improving nutritional status during hospitalization and to assess changes in blood parameters as nutritional indicators.

## Materials and methods

Study design and patient selection

This single-center, retrospective observational study was conducted at the Japanese Red Cross Kumamoto Hospital from February 1, 2020, to January 31, 2023. The hospital is an acute care facility in Japan that provides medical care for patients with a wide range of medical conditions. The study population included hospitalized patients who received acupuncture for reduced dietary intake. Reduced dietary intake was defined as an average dietary intake of less than 50% during the seven days prior to the initiation of acupuncture treatment, consistent with the etiological criteria for malnutrition outlined by the Global Leadership Initiative on Malnutrition [10]. All patients who received acupuncture for reduced dietary intake were included in the study. Patients were excluded if they did not consent to participate, if their dietary intake could not be observed for at least three days before and after the acupuncture treatment, or if they were managed with enteral nutrition. An “Explanation and Consent Form” was mailed to all eligible patients, and written informed consent was obtained. In cases where patients were unable to provide consent or had deceased, a representative was asked to sign the form on their behalf.

Ethical approval

The study was conducted in accordance with the Declaration of Helsinki and its amendments, as well as the “Guidelines for Good Clinical Practice” issued by the Ministry of Health, Labour, and Welfare of Japan for epidemiological and clinical research. The study was approved by the Ethics Review Committee of the Japanese Red Cross Kumamoto Hospital (Approval No. 543) and was registered in the University Hospital Medical Information Network (UMIN) Clinical Trials Registry (UMIN000055185).

Sample size determination

The required sample size for this study was calculated using the power analysis software G*Power (version 3.1.9.7; Heinrich-Heine-Universität, Düsseldorf, Germany). Assuming an effect size of 0.5, a significance level (α error) of 0.05, and a power (1-β error) of 0.90, the calculated sample size was 36 cases. As a pilot study, we initially sampled 36 consecutive patients who received acupuncture treatment for decreased dietary intake and observed changes in their dietary intake. Using data from these 36 cases, we calculated the population variance and maximum effect size, and applied the following formula to determine the required sample size (with α error of 0.05, β error of 0.2, allowable error (μ-μ₀) of 0.03, population variance (σ²) of 0.62, maximum effect size (δ) of 0.269): 

\[\text{N}=\tfrac{(Zα + Zβ)^2 σ^2}{(μ - μ₀ - δ)^2}\]

The calculated sample size was 68 cases. By January 31, 2023, we had identified 80 patients who received acupuncture interventions. Since this number exceeded the required sample size, we included all 80 cases in the study.

Outcome measures

The primary outcome was the rate of daily change in dietary intake percentage over the seven days before and after acupuncture treatment. Secondary outcomes included the rate of daily change in dietary intake percentage per meal before and after acupuncture treatment; changes in blood parameters - including total lymphocyte count (TLC), hemoglobin (Hb), albumin (ALB), prealbumin (PALB), and C-reactive protein (CRP) - before and after acupuncture treatment, and safety evaluations based on the presence or absence of adverse events during the treatment period.

**Figure 1:**
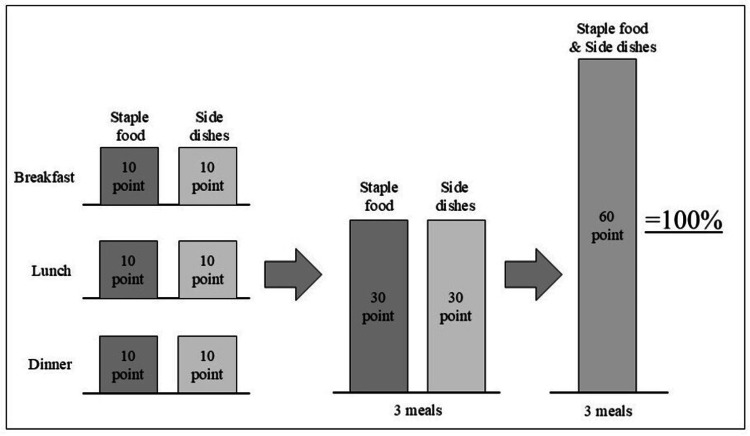
Method to calculate the dietary intake rate percentage The amount of staple food and side dishes consumed at breakfast, lunch, and dinner was recorded using a 10-point scale for each component. The maximum total score for the three meals was 60 points. The sum was divided by 60 and multiplied by 100 to calculate the daily dietary intake percentage (maximum 100%).

Dietary intake was independently assessed by nurses who recorded the amount of staple food and side dishes consumed by patients at breakfast, lunch, and dinner using a 10-point scale in the medical records. The nurses had 1-40 years of experience. Acupuncturists referred to this recorded information to confirm each patient’s daily dietary intake by summing the scores from the three meals. The maximum combined score for three meals in a day was 60; thus, the sum was divided by 60 and multiplied by 100 to determine the daily dietary intake percentage, as illustrated in Figure 1. Graphs illustrating the proportion of dietary intake were generated for the seven days before and the seven days after acupuncture treatment, and the rate of daily change in dietary intake was then calculated based on the slope of the linear regression lines. For blood parameters, due to the limited number of measurements in routine clinical practice, all data during the observation period were plotted, and the trend of change for each parameter was similarly evaluated using the slope of linear regression lines.

Patients for whom measurements were not performed during the study period were excluded from the analysis of those parameters.

Acupuncture intervention

The acupuncture treatment in this study reflects routine clinical practice. Accordingly, each patient received a fully individualized treatment based on Traditional Chinese Medicine (TCM) theory, including pattern identification and acupoint selection tailored to the specific diagnosis.

The acupuncture treatment sessions lasted 20-30 minutes, with interventions conducted once daily, five times a week (Monday to Friday, excluding Japanese holidays). When patients refused acupuncture treatment, no intervention was conducted on that day; therefore, the maximum number of treatment sessions during the observation period was five. The number of sessions each patient received is individually reported in the Results section. The acupuncture treatments were performed by a single acupuncturist certified by the Japan Society of Acupuncture and Moxibustion (JSAM), with 4-7 years of clinical experience during the study period. The treatment needles included Kozato-style Tei-shin (pure silver) (Ito Acupuncture Medical Equipment Manufacturing Company, Gifu, Japan); J15SP No. 2 (diameter 0.12 mm × length 15 mm; stainless steel), JSP No. 1 (diameter 0.16 mm × length 40 mm; stainless steel), and JSP No. 2 (diameter 0.18 mm × length 50 mm; stainless steel) (SEIRIN Co. Ltd., Shizuoka, Japan); and Pyonex needles (diameter 0.15 mm × length 0.6 mm) (SEIRIN Co. Ltd., Shizuoka, Japan). Needle insertion was performed using the Kanshin Method, with insertion depths ranging from 5 mm to 10 mm and a needle retention time of seven minutes. Manual techniques aimed at eliciting strong deqi sensations - typically characterized by a heavy feeling around the acupoint, as emphasized in TCM-style acupuncture - were not performed. Kozato-style Tei-shin provided only superficial stimulation by pressure on the skin and was primarily employed for patients susceptible to decreased neutrophil counts and infection. The JSP No. 1 was the standard-use needle, whereas the J15SP No. 2 was used for areas with heightened pain sensitivity or for tonifying purposes. JSP No. 2 was used for reduction techniques, and Pyonex needles were used for sustained stimulation of an acupoint. The electronic moxibustion device was “IKKYU” (Chuo Co. Ltd., Hyogo, Japan). No control group was included in this study. Japanese Kampo medicines were prescribed by the attending physicians during routine medical care.

Reliability of nutritional indicators

Among these, TLC and ALB are recognized prognostic biomarkers and were used to calculate the prognostic nutritional index, a marker for assessing overall nutritional status and immune function [11]. Hb levels, which are strongly associated with frailty in older adults, served as a useful biomarker for malnutrition [12,13]. PALB was also assessed as a screening marker for malnutrition during hospitalization [14]. CRP levels increased, while ALB levels decreased in response to inflammation, reflecting a negative correlation between these markers. Composite indicators, such as the Glasgow Prognostic Score and the CRP-ALB ratio (the ratio of CRP to ALB), were used as indirect markers for cancer cachexia [15].

Statistical analyses

The primary outcome was analyzed using a paired t-test. Data were presented as mean ± standard deviation. Statistical analyses were performed using the EZR software (version 1.68; Saitama Medical Center, Jichi Medical University, Saitama, Japan). P-values of < 0.05 were considered statistically significant.

## Results

Study population

**Figure 2:**
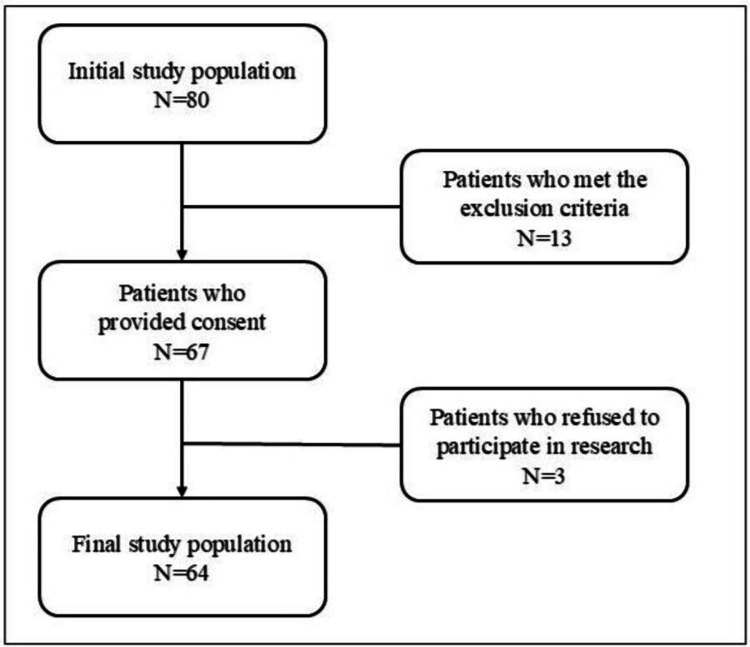
Flow diagram of the trial Patients excluded from analysis comprised of those receiving ongoing enteral nutrition or lacking dietary intake percentage data for the three days before and after acupuncture treatment. These exclusions were necessary as it was not possible to calculate the rate of daily change in dietary intake percentage for these cases.

**Figure 3:**
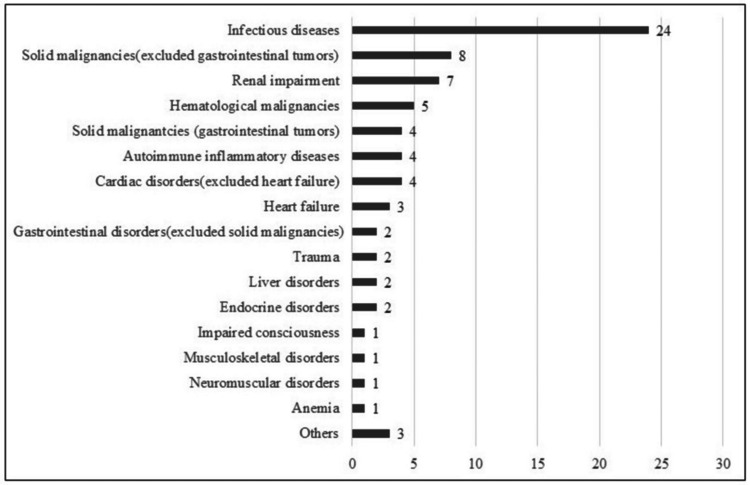
Classification of reasons for hospitalization The horizontal axis indicates the number of cases for each diagnostic category leading to hospital admission.

Of the 80 patients who received acupuncture during the study period, 13 were excluded due to ongoing enteral nutrition or incomplete dietary intake data for the three days before and after acupuncture treatment, making it impossible to calculate the daily change in dietary intake percentage. Additionally, three participants did not provide consent to participate in this study and were excluded. Ultimately, 64 participants were included in the final analysis. The patient selection process is shown in Figure 2. Among the 64 participants, 21 (33%) were male and 43 (67%) were female, with a mean age of 75.0 ± 12.7 years. The most common reasons for hospitalization included infectious diseases (24 cases; 37.5%), solid malignancies (excluding gastrointestinal tumors) (eight cases; 12.5%), and renal impairment (seven cases; 10.9%). Additional patient characteristics, including age, sex, length of hospital stay, time from admission to acupuncture intervention, number of acupuncture sessions during the observation period, total number of sessions during hospitalization, clinical outcomes, and primary diagnoses are summarized in Table 1. Figure 3 presents a categorical summary of the primary diagnoses leading to hospitalization.

**Table 1:** Patient baseline characteristics M: Male; F: Female

No.	Age (years)	Sex (M/F)	Length of hospital stay	Time from admission to acupuncture intervention	Number of acupuncture sessions during the observation period	Total number of acupuncture sessions during hospital stay	Clinical outcomes	Primary diagnoses
1	47	F	23	11	5	8	Hospital transfer	Hematological malignancies
2	82	F	17	10	2	2	Death	Solid malignancies (gastrointestinal tumors)
3	73	F	37	27	5	6	Discharge	Infectious diseases
4	91	M	19	5	5	9	Hospital transfer	Impaired consciousness
5	72	M	39	38	5	17	Discharge	Renal impairment
6	58	F	54	11	2	9	Hospital transfer	Infectious diseases
7	77	F	23	10	5	9	Hospital transfer	Renal impairment
8	83	F	19	16	3	3	Discharge	Hematological malignancies
9	69	M	39	10	5	19	Hospital transfer	Infectious diseases
10	49	F	14	40	3	3	Discharge	Renal impairment
11	67	F	18	9	3	4	Hospital transfer	Hematological malignancies
12	74	F	46	15	5	20	Hospital transfer	Solid malignancies (excluded gastrointestinal tumors)
13	92	F	19	8	3	5	Hospital transfer	Infectious diseases
14	77	F	28	15	5	8	Hospital transfer	Infectious diseases
15	76	F	26	9	5	12	Discharge	Solid malignancies (excluded gastrointestinal tumors), infectious diseases
16	78	F	17	10	3	3	Death	Anemia
17	69	M	18	8	5	8	Discharge	Others
18	69	F	54	12	5	29	Hospital transfer	Infectious diseases
19	91	F	26	10	5	9	Death	Infectious diseases, cardiac disorders (excluded heart failure)
20	70	F	154	45	5	68	Hospital transfer	Infectious diseases
21	81	M	46	36	4	8	Discharge	Cardiac disorders (excluded heart failure)
22	91	F	43	21	5	11	Hospital transfer	Infectious diseases
23	62	F	32	18	5	10	Discharge	Infectious diseases
24	43	F	176	74	5	59	Discharge	Solid malignancies (excluded gastrointestinal tumors)
25	76	M	41	13	3	17	Death	Infectious diseases, autoimmune inflammatory diseases
26	85	F	20	8	5	9	Discharge	Heart failure
27	74	M	79	10	3	44	Hospital transfer	Infectious diseases
28	68	M	54	12	4	10	Hospital transfer	Renal impairment, liver disorders
29	74	F	64	32	5	18	Hospital transfer	Cardiac disorders (excluded heart failure), infectious diseases
30	80	F	33	16	3	10	Hospital transfer	Hematological malignancies, infectious diseases
31	85	M	14	7	4	4	Discharge	Infectious diseases
32	90	F	26	17	5	6	Discharge	Others
33	86	F	36	10	5	17	Discharge	Solid malignancies (gastrointestinal tumors)
34	58	F	18	11	5	5	Death	Endocrine disorders
35	44	F	59	37	5	15	Discharge	Solid malignancies (excluded gastrointestinal tumors)
36	59	M	52	31	4	7	Hospital transfer	Trauma
37	78	F	33	39	5	9	Discharge	Infectious diseases
38	86	F	43	11	4	17	Hospital transfer	Autoimmune inflammatory diseases
39	94	F	30	22	3	4	Discharge	Infectious diseases, gastrointestinal disorders (excluded solid malignancies)
40	85	F	38	24	5	10	Hospital transfer	Trauma
41	92	M	18	10	5	6	Discharge	Infectious diseases, renal impairment
42	72	F	28	18	5	6	Discharge	Solid malignancies (excluded gastrointestinal tumors)
43	76	M	18	11	5	5	Discharge	Renal impairment
44	56	F	58	9	5	30	Death	Solid malignancies (excluded gastrointestinal tumors)
45	57	F	28	14	4	9	Death	Liver disorders
46	96	M	34	10	5	11	Hospital transfer	Infectious diseases
47	88	M	23	12	5	7	Hospital transfer	Heart failure
48	84	F	35	20	5	7	Hospital transfer	Heart failure, infectious diseases
49	91	M	33	15	2	8	Death	Endocrine disorders
50	76	M	18	14	2	2	Hospital transfer	Solid malignancies (gastrointestinal tumors)
51	61	M	64	19	5	28	Hospital transfer	Solid malignancies (excluded gastrointestinal tumors)
52	61	F	21	8	5	9	Discharge	Autoimmune inflammatory diseases
53	83	F	33	17	5	9	Hospital transfer	Neuromuscular disorders
54	83	F	15	10	3	3	Hospital transfer	Others, infectious diseases
55	93	F	46	23	5	14	Discharge	Infectious diseases
56	85	M	19	11	5	6	Hospital transfer	Musculoskeletal disorders
57	69	M	80	53	5	18	Discharge	Cardiac disorders (excluded heart failure)
58	77	F	70	24	3	3	Discharge	Gastrointestinal disorders (excluded solid malignancies
59	69	M	23	7	4	11	Discharge	Autoimmune inflammatory diseases
60	77	F	30	9	4	11	Death	Hematological malignancies
61	76	M	53	47	3	3	Death	Solid malignancies (excluded gastrointestinal tumors)
62	70	F	22	6	2	2	Discharge	Infectious diseases
63	85	F	43	9	3	9	Hospital transfer	Renal impairment
64	61	F	26	11	4	4	Discharge	Solid malignancies (gastrointestinal tumors)

Rate of change in dietary intake percentage

**Figure 4:**
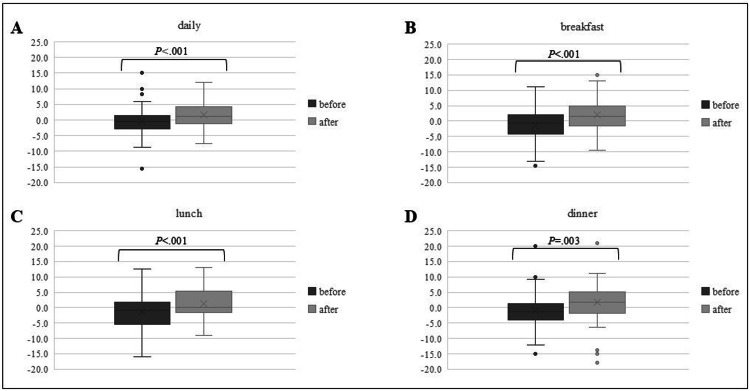
Rate of change in dietary intake before and after acupuncture treatment Box plots representing changes in (A) overall daily intake, (B) breakfast, (C) lunch, and (D) dinner. The vertical axis indicates the percentage change. The box edges indicate the 25th and 75th percentiles, the whiskers represent the 10th and 90th percentiles, and the line within each box shows the median value.

The rate of daily change in dietary intake percentage was -1.08 ± 3.99% before the initiation of acupuncture treatment; however, it significantly increased to 1.93 ± 4.10% after the treatment began (P < 0.001). Similar trends were observed across all three meals, as shown in Figure 4. The rate of daily change in dietary intake percentage increased from -1.46 ± 5.43% to 2.09 ± 5.42% for breakfast (P < 0.001), from -1.67 ± 5.00% to 1.53 ± 5.34% for lunch (P < 0.001), and from -0.70 ± 5.07% to 2.22 ± 5.88% for dinner (P = 0.003). The underlying causes of reduced dietary intake were not clearly identified.

Changes in blood parameters

Increased TLC, Hb, ALB, and PALB levels were observed in 16/48 (33.3%), 21/64 (32.8%), 24/52 (46.2%), and 4/7 (57.1%) cases, respectively. Additionally, decreased CRP levels were noted in 40/59 cases (67.8%), as shown in Table 2.

**Table 2:** Changes in blood parameters before and after acupuncture treatment Missing data are indicated by (-). TLC: Total lymphocyte count; Hb: Hemoglobin; ALB: Albumin; CRP: C-reactive protein; PALB: Prealbumin

No.	TLC (/mm^3^)	Hb (x10^4^/μL)	ALB (g/dL)	CRP (mg/dL)	PALB (mg/dL)
1	9.2	-0.2	-0.1	0.3	-
2	-	-0.2	-	-	-
3	-	0	0	0	-
4	-	0.1	0	-0.2	-
5	-	-0.3	0	0.5	-
6	152.7	-0.1	0	-0.8	-
7	86.1	-0.2	0	0	-
8	-11.1	0	-	-0.6	-
9	-8.7	-0.1	-	-	-
10	-146.4	0.1	0	-	-
11	-9.1	0	0	0	-
12	-42.6	0	-0.1	-0.1	-
13	-12.4	-0.1	0	-0.1	-
14	-	0.1	-	0.4	-
15	-10.7	0	0	0	-
16	-39.2	0.2	0	0	-
17	-7.8	-0.2	-0.1	-1.6	-
18	10.1	-0.1	0	-0.1	-
19	-19.1	-0.2	0	-	-
20	-25.7	0	0	0.6	-0.5
21	20.8	0	0.1	-0.5	-
22	-	-0.3	0	-0.7	-
23	32.9	0	0	0	-
24	-80.0	0.1	0	0.3	0.3
25	-	0	0	-1.1	-
26	35.8	0.1	0	0.3	-
27	-	0.1	0	0.1	-
28	-109.7	0	0	-0.3	0.1
29	81.5	0.1	0	-0.1	0
30	-46.1	0.1	-	0	-
31	86.9	-0.1	-0.5	0	-
32	-	-0.1	-	-0.2	-
33	-30.8	-0.1	0	-0.5	-
34	-182.6	-0.1	0	-0.3	-
35	-1.1	-0.2	-	-0.2	-
36	30.9	0.1	0	0	0.3
37	71.7	-0.2	0	-0.3	-
38	-	0.1	0.1	-0.2	-
39	-19.6	-0.1	0	0.1	-0.1
40	-	0	0	0.7	-
41	-	0	0	-0.4	-
42	-6.0	0.2	-	-1.2	-
43	42.5	0	0	-0.8	-
44	-26.3	0	0	-0.3	0.8
45	-	0.1	-0.1	-	-
46	-39.9	0	0	0.1	-
47	3.6	0.1	-	-0.4	-
48	-195.6	-0.1	0	-1.4	-
49	-48.8	0	-0.9	0	-
50	-38.2	0	-	-0.4	-
51	-26.6	-0.3	-0.1	-1.1	-
52	-27.0	0	0	0	-
53	31.5	0	0	-0.4	-
54	-166.5	-0.1	-0.1	-0.5	-
55	-	0.1	0	-0.1	-
56	-94.1	-0.1	-	2.2	-
57	-	-0.1	-	0.3	-
58	-323.7	0.1	0	0.3	-
59	25.8	0	0	0	-
60	-2.4	0	0	-0.4	-
61	-	0	0	0.8	-
62	-29.2	0	0	-2.0	-
63	5.7	0	0	0	-
64	-37.2	-0.1	-0.1	0.1	-
Effective	16	21	24	40	4
Cases (n)	48	64	52	59	7
Effective rate (%)	33.3	32.8	46.2	67.8	57.1

Concurrent use of Japanese Kampo medicines

**Figure 5:**
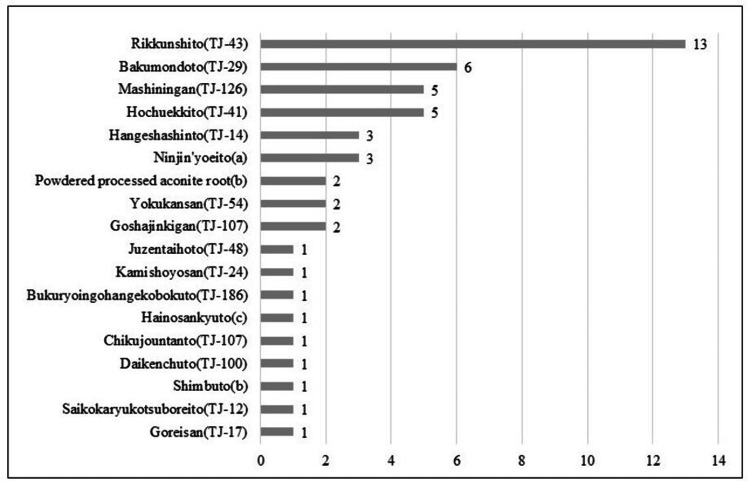
Frequency of combined Japanese Kampo medicine usage The horizontal axis represents the number of cases for each Kampo formulation. Formulation manufacturers are indicated as follows: (a) Kracie Pharmaceutical Co. Ltd., Tokyo, Japan; (b) Sanwa Shoyaku Co. Ltd., Wakayama, Japan; (c) Kotaro Pharmaceutical Co. Ltd., Osaka, Japan.

In total, 41/64 patients (64.1%) received Japanese Kampo medicines concurrently with acupuncture treatment, of which one patient was administered three different types, seven patients received two types, and 33 patients received a single type. The rate of dietary intake improvement was 59.1% in patients who received acupuncture alone, which increased to 73.8% in those who were treated with both acupuncture and Japanese Kampo medicines. The most frequently prescribed Japanese Kampo medicines were Rikkunshito (TJ-43) (13 cases), Bakumondoto (TJ-29) (six cases), Mashiningan (TJ-126) (five cases), and Hochuekkito (TJ-41) (five cases), as shown in Figure 5.

Acupoint usage frequency

**Figure 6:**
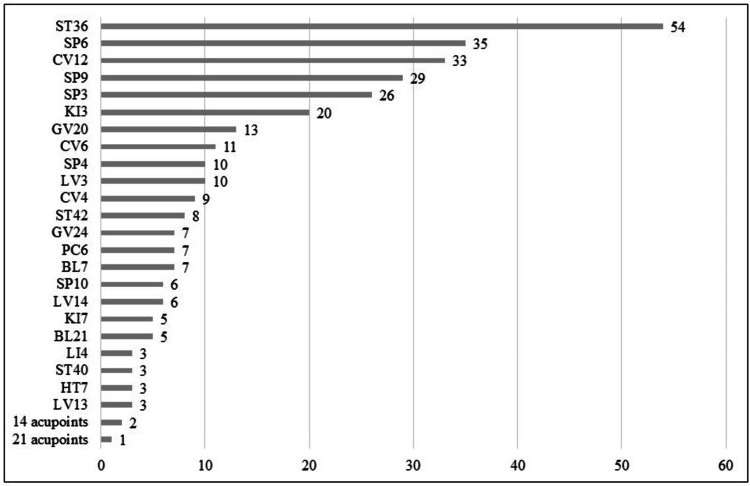
Frequency of acupoint usage The horizontal axis represents the number of cases. The group of 14 acupoints includes CV22, ST9, ST21, BL10, BL17, BL23, BL60, GB20, TE4, LV4, Hx-HN1, Ex-B7, shenmen (auricular acupoint), and sympathetic (auricular acupoint). The group of 21 acupoints includes CV23, LU1, LU7, LU9, ST25, SI13, BL2, BL25, BL57, PC7, TE5, TE23, GB1, GB34, GB37, GB42, GB43, Ex-HN3, Ex-B2, brainstem point (YNSA), and basic D point (YNSA). YNSA: Yamamoto new scalp acupuncture

An average of 7.14 ± 1.50 acupoints were used per treatment session. Acupoints used in > 50% of the sessions during the observation period were considered to have a high-use frequency. The top three high-use frequency acupoints were ST36 (54 cases), SP6 (35 cases), and CV12 (33 cases), as shown in Figure 6.

Safety assessment

No serious adverse events requiring medical intervention, such as organ damage, were observed in association with acupuncture treatment. Although minor bleeding was noted at the time of needle removal in some cases, these events resolved spontaneously and did not necessitate clinical management. Furthermore, no cases of refeeding syndrome were reported following increased dietary intake.

## Discussion

To the best of our knowledge, this study is the first to examine the effects of acupuncture on reduced dietary intake in hospitalized patients. The daily dietary intake rate showed a declining trend before acupuncture treatment; however, it significantly improved within seven days after treatment initiation. Since no new medical interventions were introduced during this period and rapid improvement was observed immediately after starting acupuncture or acupuncture combined with Japanese Kampo medicines, these findings suggest that the observed effects were attributable to these interventions rather than natural recovery.

The dietary intake rate significantly improved across breakfast, lunch, and dinner. Fluctuations in meal intake are often associated with circadian rhythm disturbances, which can disrupt the normal sleep-wake cycle and suppress appetite at specific times of the day [16]. However, in this study, the rate of increase in dietary intake remained consistent across all meals. These findings suggest that acupuncture, alone or in combination with Japanese Kampo medicines, may help enhance the overall rate of dietary intake in patients with a well-functioning circadian rhythm.

The observation period for blood markers in this study was short, which may explain the lack of significant changes in the blood test results during the study period. CRP, an inflammatory marker, can fluctuate over short periods and may be influenced by new infections during hospitalization. PALB, a precursor of ALB and a nutritional marker, has a short half-life of 2-3 days [17], making it sensitive to early dietary changes. In our study, PALB was measured in a limited number of participants (seven individuals), of whom four (57%) showed an increase. This suggests that the increase in dietary intake may have contributed to this change. To clarify this relationship, it is necessary to conduct further investigations with a larger sample size.

The rate of change in dietary intake was higher when acupuncture was combined with Japanese Kampo medicines (73.8%) compared to acupuncture alone (59.1%). In total, 64.1% of the patients received Japanese Kampo medicines, with Rikkunshito (TJ-43) being the most prescribed. The flavonoids in Rikkunshito (TJ-43) elicit 5-hydroxytryptamine 2B receptor and 5-hydroxytryptamine 2C receptor antagonistic effects, promote ghrelin release, improve gastrointestinal dysfunction, and enhance dietary intake and appetite [18,19]. These findings suggest that combining acupuncture and Japanese Kampo medicines may have a synergistic effect in enhancing dietary intake.

The most frequently used acupoints were primarily those associated with the stomach and spleen meridians, which correspond to the digestive system in Japanese Kampo medicine. Additionally, several acupoints, including GV20, CV17, GV24, PC6, and HT7, were employed to address psychological symptoms such as hypoactive delirium. Therefore, the observed increase in dietary intake may have helped improve hypoactive delirium.

In our study, no serious adverse events due to acupuncture treatment, such as organ damage, were observed, and refeeding syndrome associated with increased dietary intake did not occur, confirming the safety of the treatment. A prospective study by Furuse et al. [20] evaluated the safety of acupuncture, reporting that 6.3% of the participants experienced adverse events. However, these were primarily mild and transient and included subcutaneous bleeding, hematoma, discomfort, and residual pain at the insertion site, with no reports of severe complications like infections or organ damage.

On average, 7.14 acupoints were used per session, and 14 needles were required when symmetrical acupoints were included. With each needle costing $0.10 (USD), the cost per acupuncture session was $1.40 (USD). However, at the hospital conducting the study, patients were not charged for acupuncture treatment.

No other medical interventions aimed at improving dietary intake were implemented, and nutritional counseling was not conducted due to factors such as advanced age and impaired consciousness. Furthermore, patients were not informed that the intervention was intended to improve nutritional status in order to minimize any placebo effect on appetite improvement. However, due to the absence of a control group, potential confounding factors cannot be ruled out.

Study limitations

This study had several limitations. First, this was a single-arm comprehensive survey without a control group. Therefore, we could not establish a causal relationship between the observed improvements in dietary intake, changes in blood test markers, and acupuncture, nor determine whether these improvements were solely due to the natural course of disease treatment. Second, although the combination of Japanese Kampo medicines and acupuncture resulted in a higher rate of dietary intake improvement than acupuncture alone, we did not evaluate the effects of Japanese Kampo medicines independently. Thus, whether the observed improvements were attributable to Japanese Kampo medicines remains unclear. Third, using different dietary intake evaluators on different days helped minimize bias. However, the absence of a sham acupuncture group means that the possibility of a placebo effect cannot be excluded. Fourth, a longer observation period is required for all blood test markers, except for PALB, to account for their half-lives. However, due to the high-acuity nature of the hospital, the relatively short duration of hospital stays made extended observation challenging.

## Conclusions

In this single-arm case series, we found that dietary intake significantly improved following acupuncture treatment alone as well as in combination with Japanese Kampo medicines; however, improvements in blood parameters were limited, and increasing the number of cases for statistical analysis is necessary. A prospective comparative trial is needed to confirm the efficacy of acupuncture in enhancing dietary intake. Moving forward, we aim to standardize acupuncture treatment protocols based on the frequently used acupoints identified in this study. Additionally, we plan to establish an effective acupuncture treatment approach for insufficient dietary intake through a prospective comparative trial with a separate control group.
